# The complete chloroplast genome of *Atraphaxis jrtyschensis* (polygonaceae), an endemic and endangered desert shrub to Xinjiang, China

**DOI:** 10.1080/23802359.2018.1508380

**Published:** 2018-09-22

**Authors:** Qiuyan Wang, Zhenyan Yuan, Yue Zhang, Sulayman Mamtimin, Xinmin Tian

**Affiliations:** a Institute of Arid Ecology and Environment, Xinjiang University, Urumqi, Xinjiang, China;; b College of Life Science and Technology, Xinjiang University, Urumqi, Xinjiang, China

**Keywords:** *Atraphaxis jrtyschensis*, chloroplast, genome, conservation genetics

## Abstract

*Atraphaxis jrtyschensis* (Polygonacae) is an endangered desert shrub endemic to China in Xinjiang province with great ecological importance for sand fixation. However, its genomic resources are still very limited. Here, we generated the first chloroplast (cp) genome of *A. jrtyschensis* using genome skimming sequencing. The whole cp genome is 164,192 bp and comprises 130 genes, including 83 protein-coding genes, 37 tRNA genes, 8 rRNA genes, and 2 pseudogenes (*rpl*23). The overall GC content of *A. jrtyschensis* cp genome is 37.5%. The phylogenic analysis placed *A. jrtyschensis* at the base of Trib Rumiceae, which contained the genera *Rheum* and *Oxyria*. This study will be useful for future researches to investigate the conservation genetics and potential applications in sand fixation of the endangered desert shrub.

*Atraphaxis jrtyschensis* Yang et Han (Polygonaceae) is an endangered desert shrub, which is endemic in the Buerjin County in Xinjiang province of Northwest China (Feng et al. 2003). It is an ecologically and economically important shrub for sand fixation and forage. However, the wild resources of *A. jrtyschensis* have been decreased dramatically and only few populations can be found in the field. It is now necessary to take effective methods to conserve this desert shrub. As far as we know, there is no study that has been focused on this shrub except a phylogenetic study of trib Atraphaxideae based on cp DNA fragments (Sun and Zhang 2012). Comprehensive genomic resources are absent for *A. jrtyschensis*, which limits the understanding of its conservation genetics at the genome level. In this study, we sequenced, assembled, and annotated the complete cp genome of *A. jrtyschensis* based on the sequencing data.

The fresh leaves of *A. jrtyschensis* were collected from the Buerjin County (86.867694E, 47.695674 N) and vouchers were deposited in Xinjiang University (Accession Number ML0025). Total genomic DNA were extracted with the CTAB method (Doyle and Doyle 1987). The complete-genome sequencing was conducted with 150 bp pair-end (PE) reads on the Illumina Hiseq Platform(Illumina, San Diego, CA). In total, 5.0 GB raw data was obtained and approximately 10 million high quality clean reads were collected after filtering low-quality reads. The complete circular cp genome were assembled using the softwere Velvet (Zerbino and Birney 2008). After manual adjustment, we performed an automated genome annotation with a recently developed command-line application called Plastome Annotator (Plann) (Huang and Cronk 2015). Then, we got a physical map using the software OGDRAW (Lohse et al. 2013). A maximum likelihood (ML) tree was reconstructed using the software RaxML version 8 (Stamatakis 2014) from the alignments created by the MEGA6.06 (Tamura et al. 2013). Finally, the complete chloroplast genome sequence and its annotations were submitted to GenBank with an accession number of MG878984.

The chloroplast genome of *A. jrtyschensis* showed a typical quadripartite structure with a length of 164,192 bp, which contained inverted repeats (IR) of 24,152 bp separated by a large single-copy (LSC) and a small single copy (SSC) of 88,878 bp and 27,010 bp, respectively. The cpDNA contains 130 genes, comprising 83 protein-coding genes, 37 tRNA genes, 8 rRNA genes, and 2 pseudogenes (*rpl*23). Among the annotated genes, 10 of them contained only 1 intron (*rps*12, *trn*K-UUU, *rps*16, *trn*G-UCC, *atp*F, *rpo*C1, *trn*V-UAC, *pet*B, *pet*D, and *ndh*A), and 5 genes (*trn*A-UGC, *trn*I-GAU, *rp*l2, *ndh*B, and *clp*P) contained 2 introns. The overall GC content of the plastome is 37.5%, while the corresponding values of the LSC, SSC, and IR regions are 35.5, 33.0, and 43.6%, respectively. Further, the phylogenetic analysis of 20 plastid genomes with *Forsythia suspensa* used as the outgroup showed that *A. jrtyschensis* is related to the Trib Rumiceae, which includes the genera *Rheum* and *Oxyria* (Figure 1). The *A. jrtyschensis* genome will be a valuable genetic resource and can be used for population genetic and phylogenetic studies of *A. jrtyschensis*.

**Figure 1.**
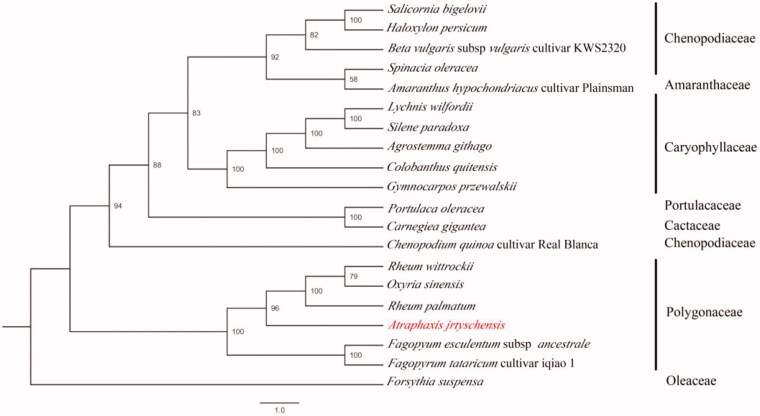
The phylogenetic tree based on seven complete chloroplast genome sequences. Accession numbers: *Rheum palmatum* (KR816224), *Rheum wittrockii* (KY985269), *Oxyria sinensis* (KX774248), *Fagopyrum tataricum* (KX085498), *Faopyrum esculentum* (EU254477), *Forsythia suspense* (MF579702), *Chenopodium quinoa* (CM008430), *Haloxylon persicum* (NC027669), *Spinacia oleracea* (AJ400848), *Beta vulgaris* (KR230391), *Amaranthus hypochondriacus* (NC030770), *Salicornia bigelovii* (NC027226), *Portulaca oleracea* (NC036236), *Carnegiea gigantean* (NC027618), *Colobanthus quitensis* (NC028080), *Silene paradoxa* (NC023360), *Agrostemma githago* (NC023357), *Gymnocarpos przewalskii* (NC036812), *Lychnis wilfordii* (NC035225).
